# Coping and Beliefs as Predictors of Functioning and Psychological Adjustment in Fibromyalgia Subgroups

**DOI:** 10.1155/2022/1066192

**Published:** 2022-04-14

**Authors:** Laura Rubio Fidel, Azucena García-Palacios, Rocío Herrero, Guadalupe Molinari, Carlos Suso-Ribera

**Affiliations:** ^1^Consorcio Hospitalario Provincial de Castelló, Castelló de la Plana, Spain; ^2^Department of Basic Psychology, Clinic and Psychobiology, Universitat Jaume I, Castelló de la Plana, Spain; ^3^CIBEROBN Physiopathology of Obesity and Nutrition, Instituto de Salud Carlos III, 28029 Madrid, Spain; ^4^Polibienestar Research Institute, University of Valencia, Valencia 46022, Spain; ^5^Valencian International University-VIU, Valencia, Spain

## Abstract

**Objectives:**

Research has pointed to two profiles of persons with fibromyalgia according to differences in functionality, thus distinguishing between functional and dysfunctional patients. The role of psychological factors underlying such clusters is unclear. This study aims to explore the contribution of pain beliefs and coping on fibromyalgia clustering.

**Methods:**

A cluster analysis was performed to classify 238 women with fibromyalgia using the Fibromyalgia Impact Questionnaire and the Beck Depression Inventory as clustering variables. Cluster differences in physical functioning, depression, pain beliefs, coping, and age were then calculated (Student's *t*-test). Finally, a binary logistic regression was conducted to study the unique contribution of age, beliefs, and coping on cluster classification.

**Results:**

Two clusters were revealed. Cluster 1 had a poor adaptation to fibromyalgia regarding physical functioning and depression. They generally embraced less adaptive beliefs (i.e., disability, harm, emotion, and requests) and coping strategies (i.e., guarding, resting, and asking for assistance). Cluster 2 showed a better adaptation to fibromyalgia and adopted more favorable beliefs (i.e., control) and coping strategies (i.e., exercise and task persistence). Cluster differences in age were significant but small. The backward binary logistic regression suggested a final model with six predictors (guarding, task persistence, harm, emotion, solicitude, and age) that explained 31% of the variance of group membership. *Discussion*. These results suggest that only a subset of psychological variables uniquely and independently contribute to functional/dysfunctional group membership. The results support the need to address psychological components in the management of fibromyalgia and point to a subset of preferred target beliefs and coping strategies.

## 1. Introduction

According to the 2016 revision of the 2010/2011 American College of Rheumatology (ACR) criteria, fibromyalgia syndrome (FMS) is characterized by the presence of generalized pain (defined as pain in at least 4 of 5 regions), a minimum duration of 3 months of that pain, and a combination of widespread pain and sufficient symptom severity [1, 2]. While this renewed definition of FM has been overall well accepted in the scientific community, there is also a growing trend among FM experts to consider the disease as a complex spectrum of syndromes and symptoms rather than a discrete and homogeneous clinical entity [3–5].

Certainly, patients with FMS present heterogeneous clinical manifestations and individual differences in functionality, which make it hard to understand the syndrome and to put into practice adequate therapeutic approaches [6]. To address this issue, researchers have tried to identify subgroups of patients based on their differential adaptation to the disease, generally using cluster analysis [7–10]. This method analyzes the degree of similarity between a set of heterogeneous variables with the aim to identify related groups of individuals based on their similarities in the included variables, which might ultimately help guide interventions in a more effective and personalized manner [11].

De Souza and colleagues were the first to attempt to classify persons with FMS according to their functional status [12]. Using the Fibromyalgia Impact Questionnaire (FIQ), a gold-standard measure to assess functional impairment in fibromyalgia patients [13, 14], the authors obtained two subgroups of patients. One was characterized by low anxiety, low depressed mood, and low morning tiredness symptoms. The other presented elevated pain severity, fatigue, morning tiredness, stiffness, anxiety, and depressive symptoms. Further research has supported a comparable distribution of approximately 50% of the population in each group [15].

Since the inspirational work by De Souza et al. [12], several FMS studies using cluster analysis have been published, generally using the FIQ as a grouping variable [16–18]. These studies have generally replicated this idea that there are two different adaptation profiles in response to this complex syndrome. While this might be important as a first step to improve multidisciplinary treatments for FMS, exploring the mechanisms underlying such differences (i.e., why some individuals perform better than others in front of a disease) is a necessary next step [3]. Specifically, if group differences are found in the mechanisms used to adapt to the disease, these could potentially serve as important therapeutic goals, especially for the dysfunctional group.

There is extensive literature into psychological mechanisms associated with the adaptation to FMS. For example, catastrophizing, fear of movement, and activity avoidance, which are key factors in the fear-avoidance model of pain [19], have repeatedly predicted greater disability, distress, and interference of this condition with daily life [20–24]. According to this model, maladaptive cognitive appraisals in the presence of pain (e.g., catastrophizing and excessive threat appraisal) would lead to maladaptive coping efforts (e.g., avoidance of activities and hypervigilance) and therefore to disuse, disability, and depression, which negatively contribute to FMS [25–27]. As noted by the same authors of the fear-avoidance model of pain, pain is a threatening, interfering, and stressful experience [28], which certainly provides an appropriate context in which to consider the role of beliefs, which are assumptions about reality which serve as a perceptual lens through which events are interpreted, and coping, which have been defined as efforts used to deal with situations where a person thinks that the demands of the situation exceed the perceived resources [29].

In addition to beliefs and coping strategies, personality characteristics, such as the tendency to worry, experience intense and frequently changing negative emotions, and view the world as threatening (i.e., neuroticism) and harm-avoidance personality styles, have also been found in persons with FMS and are associated with poorer adaptation to the disease [30–33]. Again, these studies point avoidance behavior and harm beliefs as potentially maladaptive forms of dealing with FMS.

While the previous list of individual differences associated with the adaptation to FMS is far from complete, what studies appear to suggest is that, in the presence of a stressor like chronic pain, certain appraisals about the pain experience and coping efforts to deal with it will be more adaptive than others. For example, catastrophizing about the pain, exaggerating its threat value, and believing that there is nothing that one can do to deal with the situation (e.g., low self-efficacy) are likely to lead to maladaptive coping efforts (e.g., avoidance and impulsive solutions). On the contrary, perceiving that one has some control over the situation (i.e., the pain), that there is no need to escape it, and being willing to experience pain is generally associated with more adaptive, flexible, and rational coping [16, 34–36].

Drawing on previous work, the goal of the present study was to investigate individual differences in the psychological mechanisms used to adapt to FMS between individuals with a poor and an optimal physical and mental adaptation to the syndrome. Based on the previous literature search and in line with the fear-avoidance model of pain [19], both behavioral and cognitive factors (i.e., coping and beliefs) were selected as potentially important mechanisms that might underlie FMS clustering and therefore explain individual differences in the adaptation to the disease. According to previous research, we expect that coping strategies (e.g., guarding, resting, and asking for assistance) and pain beliefs (e.g., “hurt signifies physical injury”) that are avoidant and threat-related will be more present in patients that belong to the dysfunctional group. On the contrary, we anticipate that coping strategies like “persisting in a task” and beliefs such as “perceiving that one has control over pain,” which are more positively oriented towards the continuation of important life objectives, will be more frequent in the functional group [37–39].

Because psychological mechanisms have communalities [40], we also hypothesize that only a subset of mechanisms will be uniquely associated with belonging to the dysfunctional/functional group when explored in a multivariate manner due to shared variance. In addition, because depression is very frequent in people with fibromyalgia [41] and its evaluation with the FIQ using a single item (“Please rate your level of depression”) is debatable [42], another contribution of the present investigation will be to include a formal and widely used measure of depression for clustering, that is, the Beck Depression Inventory-II [43].

## 2. Materials and Methods

### 2.1. Participants and Procedures

Participants were 238 women with FMS. Participants were patients seeking assistance at the Psychology Clinic at Jaume I University, which offers specific psychological treatment for chronic pain and other populations. This study is part of a funded project that was approved by an ethical review board.

All patients had a main diagnosis of FMS made by a rheumatologist at the Hospital General de Castellon. Participants were referred from public and private rheumatology services, pain clinics, and FM patient associations. The data presented here come from baseline evaluations (screening sessions), before a treatment plan was proposed or patients were referred to other centers.

To participate in this cross-sectional study, FMS patients had to be over 18 years of age, present a main diagnosis of FMS provided by a rheumatologist, and give informed consent to use their assessment data for research purposes. The exclusion criteria included suffering from a severe mental disorder (psychosis or bipolar disorder), presenting suicide risk, or having a physical illness that would interfere with participation into the study. All participants were interviewed by a psychologist with expertise in the psychological treatment of chronic pain to explore these criteria.

### 2.2. Measures

#### 2.2.1. Fibromyalgia Impact Questionnaire (FIQ) [44, 45]

The FIQ is a self-administered questionnaire that measures components of health affected by FM. It is considered to be the gold-standard measure to assess functional impairment in persons with FMS. The assessment is conducted through six domains including pain, tenderness, fatigue, stiffness, multidimensional function, and sleep. Scores are interpreted on a scale ranging from 0 to 10, where higher scores represent the greater impairment.

#### 2.2.2. Beck Depression Inventory-II (BDI-II) [43, 46]

The BDI is a 21-item questionnaire designed to measure the intensity and severity of current depressive symptoms. The items are answered in multiple-choice Likert scale ranging from 0 to 3. Each item has a different response label. The total scale score ranges from 0 to 63, where 0–13 indicate the minimal depression, 14–19 indicate the mild depression, 20–28 indicate the moderate depression, and 29–63 indicate the severe depression. A higher score indicates more severe depressive symptomatology.

#### 2.2.3. Chronic Pain Coping Inventory (CPCI-42) [47, 48]

The CPCI-42 is the abbreviated version of the original 65-item Chronic Pain Coping Inventory (CPCI), an instrument that evaluates the cognitive and behavioral pain coping strategies used for pain management in the last week. These strategies are grouped into eight categories, namely, guarding (to restrict the movement or general use of a body part), resting (e.g., to lie down or sit down), asking for assistance (request help from someone for a task), relaxation (do a specific relaxation activity or one that helps you to relax, e.g., think of something pleasant), task persistence (continue to perform an activity despite being in pain), exercise/stretch (do stretching or general physical exercise), seeking social support (to talk or relay on a friend or relative), and coping self-statements (evaluate the problem of pain positively or negatively). Items are scored from 0 days to 7 days depending on the number of days each coping strategy is used. A higher score means more frequent use of the strategy in the past week.

#### 2.2.4. Survey of Pain Attitudes-25 (SOPA-25) [49, 50]

The SOPA is a 25-item measure of pain-related attitudes and beliefs. These are grouped in seven subscales. Some reflect maladaptive beliefs, such as “disability” (to which degree they believe that their functioning is impaired due to pain), “harm” (to what extent they identify pain as a sign of self-harm and should therefore avoid such activity), “medication” (to what extent they believe that medication is a good option as a treatment for chronic pain), “solicitude” (how far they think that other people, specially relatives, should pay attention to their experience of pain), and “medical cure” (the extent to which patients believe that medicine or medical cures can help with their pain). Other scales, such as “control” (the degree to which they believe they can control their pain) and “emotion” (to what extent they believe their emotions influence the experience of pain), reflect adaptive beliefs. Respondents are asked to indicate their degree of agreement using a 5-point Likert scale ranging from 0 (very false for me) to 4 (very true for me). A higher score on a subscale implies a greater degree of certainty about the corresponding belief or pain-related attitude.

We used the Spanish adaptations of all the questionnaires, which have obtained excellent reliability results in past similar research [45, 46, 48, 50].

### 2.3. Data Analysis

All analyses were conducted using SPSS version 26 [51].

First, a descriptive analysis of demographic and study variables was performed. A two-step cluster analysis was then conducted to check whether the clinical subgroups based on functionality found in previous studies were replicated. In the cluster analysis, we used a Bayesian criterion for grouping purposes. Based on past research, scores on the FIQ-R were used for grouping. Besides, the BDI scores were added to the clustering procedure in the present study because this is a more robust measure of the emotional status of individuals. Both variables were standardized using z scores because they were measured on a different scale.

In the cluster analysis, variable importance, which ranges from 0 (“no importance”) to 1 (“maximum importance”), was used as an index to evaluate whether both FIQ-R and BDI contributed to clustering. In addition, the quality of the cluster, which ranges from −1 (very poor) to 1 (good), and the ratio of sizes, which evaluates the ratio between the largest and the smallest cluster, were also used as indicators of the quality of the clusters. Regarding the latter, very large ratios indicate that at least one cluster is very rarely represented [52]. In our cluster analyses, the quality of the cluster was in the good range (0.6), both the FIQ-R and BDI contributed to clustering (variable importance values above 0.7), and ratio of sizes was excellent (1.04); further analyses were conducted with the results of this clustering.

Next, cluster subgroups were compared in the FIQ, depression, beliefs, coping, and age using Student's *t*-test to evaluate differences in study variables. Age was included to explore whether this could be a covariate in further analyses [53]. Differences in marital status and educational level were also investigated as potential covariates by means of a chi-square test.

As a final step, a binary logistic regression was conducted to study the unique contribution of each coping and belief variable on cluster classification when controlling for shared variance. Similar to past research [54], a backward method was used because this method allows obtaining more parsimonious models by excluding variables based on changes in likelihood.

## 3. Results

The age of the participants ranged from 23 to 77 (mean = 46.60 years; SD = 9.82). In total, 64.01% of them were in a relationship at the time of assessment. The remaining participants were separated (15.8%), widowed (2.1%), or single (17.9%).

Regarding the educational level, 32.4% of the participants had no studies or primary studies only, 34.9% of them had completed secondary studies, and 32.7% had completed technical or university studies.

As given in Table 1, the cluster analysis revealed two clusters, which differed in their adaptation to FMS according to the *t-*tests. The quality of the clusters was good (average silhouette = 0.6).

Cluster 1 (122 patients, 51.3%) included participants with a poorer adaptation to FMS in terms of fibromyalgia impact on functioning (mean = 74.41; SD = 10.40) and depression (mean = 31.16; SD = 7.99). Cluster 2 (116 patients, 48.7%) included patients characterized by better adaptation to the syndrome (FIQ: mean = 50.72, SD = 15.50; BDI: mean = 14.73; SD = 6.03). Differences in FM impact and depression were significant (*t* = 13.77, *p* < 0.001, *d* = 1.79 and *t* = 17.96, *p* < 0.001, *d* = 2.32, respectively) and large.

According to the BDI-II cutoffs, average depression levels in cluster 1 would correspond to severe depression, while those of cluster 2 would correspond to mild depression.

In terms of age, the differences were significant (*t* = −2.44, *p*=0.015, *d* = 0.31) but small. Individuals in cluster 1 were slightly younger (mean = 45.11, SD = 9.48) than participants belonging to cluster 2 (48.20, SD = 9.97). Marital status (*χ*^2^ = 0.585, *p*=0.444) and educational level (*χ*^2^ = 2.035, *p*=0.362), which were other potential covariates of functional status, were comparable across clusters.

Taking coping variables, significant differences were found in guarding (*t* = 4.55, *p*=<0.001, 95% CI = (0.54, 1.37), *d* = 0.59), resting (*t* = 2.41, *p*=0. 017, 95% CI = (0.10, 0.98), *d* = 0.31), asking for assistance (*t* = 2.65, *p*=0.009, 95% CI = (0.14, 0.95), *d* = 0.34), task persistence (*t* = −4.53, *p*=<0.001, 95% CI = (−1.60, −0.62), *d* = 0.58), and exercise (*t* = −2.45, *p*=0.015, 95% CI = (−1.27, −0.13), *d* = 0.32). When in pain, patients belonging to cluster 1 were more likely to restrict movement of the body or a body part (guarding), sit or lie down (resting), and ask for more help to continue to perform (asking for assistance) and were less likely to exercise and to continue performing when pain increased (task persistence).

Significant differences in beliefs emerged in control (*t* = −2.28, *p*=0.023, 95% CI = (−0.53, −0.04), *d* = 0.30), disability (*t* = 4.05, *p*=<0.001, 95% CI = (0.25, 0.72), *d* = 0.53), harm (*t* = 4.25, *p*=<0.001, 95% CI = (0.31, 0.85), *d* = 0.54), emotion (*t* = 3.47, *p*=0.001, 95% CI = (0.20, 0.71), *d* = 0.45), and request (*t* = 4.45, *p*=<0.001, 95% CI = (0.34, 0.88), *d* = 0.58). Compared to cluster 2, individuals in cluster 1 were more likely to believe that they lacked control over their pain (control), that their functionality was affected by the pain (disability), that they should interpret painful activities as harmful and avoid them (harm), that their emotions influence their pain (emotion), and that the people around them (family members and loved ones) should help them with their pain (request).

As given in Table 2, the backward binary logistic regression suggested a final model with six predictors, namely, guarding (*B* = −0.30, *p*=0.004, 95% CI = (0.61, 0.91)), task persistence (*B* = 0.18, *p*=0.031, 95% CI = (1.02, 1.41)), harm (*B* = −0.41, *p*=0.009, 95% CI = (0.49, 0.90)), emotion (*B* = −0.35, *p*=0.047, 95% CI = (0.50, 1.00)), solicitude (*B* = −0.32, *p*=0.044, 95% CI = (0.53, 0.99)), and age (*B* = 0.04, *p*=0.029, 95% CI = (1.00, 1.07)). The explained variance of this final model ranged from 23.1% (Cox and Snell *R*^2^) and 30.9% (Nagelkerke *R*^2^). The initial model with all the predictors only explained an additional 3% of variance compared to this parsimonious 6-factor model.

The percentage of correct clusters explained in the last step (with all six predictors) was 71.0%, while in the initial model with all variables, it was 73.6%. This implies a very low loss of classification ability and a good percentage of correct classifications for the two clusters in the last model (72.5% of correct classifications in cluster 1 and 69.4% of correct classifications in cluster 2).

## 4. Discussion

The main objective of this study was to identify subgroups of individuals with fibromyalgia based on their functionality and depression levels and to explore whether individual differences in coping and beliefs predicted subgrouping status. For this purpose and consistent with past research [16–18], a cluster analysis was performed with the FIQ. New to the literature, we included BDI-II as a grouping variable to account for the mental well-being of the participants when describing their functional status. In addition, a contribution to past research is the incorporation of measures of beliefs and coping to assess whether FM subgroups differed in psychological mechanisms that could potentially be used to guide interdisciplinary interventions in a more effective way. Overall, our analyses replicated the two clusters found in the literature (a functional group and a dysfunctional group). Interestingly, the inclusion of the measure of depression also evidenced between-group differences in mental health in addition to overall functioning status. Also, adding up to previous research, we found significant differences in the use of certain coping strategies and beliefs between the two groups. As anticipated, only a subset of these psychological variables uniquely contributed to patient classification (functional vs. dysfunctional). These results are consistent with our hypotheses and support the need to include psychological interventions in the management of FM, preferably and primarily targeting a subset of beliefs and coping strategies over others.

Our cluster analyses with measures of overall functioning despite the disease and depression levels evidenced two profiles of persons with FMS. These results are consistent with the literature findings [55], with the novelty of accounting for the emotional impact of the disease in more detail in the present investigation. The two groups had a comparable distribution in terms of frequency (almost 50% of individuals in each group), which is again consistent with previous studies [15]. The first group presented greater impact of fibromyalgia and greater severity of the depressive symptoms, and these differences were large in size when compared with the second group. In fact, the depression levels in the second group were only mild, while they were severe in the first group. This adds to the literature on functionality in persons with FM and supports the idea that approximately half of the persons with this disease will present adaptation problems (both mental and physical) due to FMS. It is important to note that, when using different clustering variables, studies have sometimes reported 4 subgroups of patients with FMS (e.g., maladaptive, adaptive, vulnerable, and resilient), but again adaptation problems would be present in approximately half of the population [56].

A key contribution of the present study was the inclusion of two psychological mechanisms that might underlie the individual differences in adaptation to the disease, namely, beliefs and coping strategies. The inclusion of these potential underlying mechanisms that explain individual differences in functional status is a recommended practice, but rare in the literature [57, 58]. Our results showed significant differences in both psychological mechanisms (i.e., beliefs and coping) between the two groups. The differences revealed were consistent with our prediction that certain coping strategies and beliefs would be more adaptive and therefore more representative of the high-functioning group or cluster 2 (i.e., control, task persistence, and exercising), while others would be more maladaptive and more frequent in the low-functioning group or cluster 1 (i.e., disability, harm, request, guarding, resting, and asking for assistance). Overall, these differences may be a good starting point when guiding interdisciplinary treatments for persons with FM.

In relation to these mechanisms, only one unexpected result was obtained. The emotion subscale of the SOPA-25 (i.e., the belief that emotions influence the experience of pain, with items such as “stress in my life increases my pain” or “there is a strong connection between my emotions and my pain level”) was more frequently adopted by patients in the lower functioning group (cluster 1). One possible explanation for this unexpected result is that, as shown in a recent study [59], the association between emotions and pain could lead patients with poorer functioning and greater emotional maladjustment to use suppressive strategies when faced with the appearance of certain emotions such as anxiety, stress, or sadness. This might, in turn, lead to greater emotional distress and a greater overall impact of fibromyalgia on quality of life.

Research has indeed indicated emotion regulation difficulties in persons with FMS, such as emotional suppression and rejection of emotions, and has pointed to these forms of emotion regulation as predictors of worse outcomes in this population [59–61]. Other difficulties in the first steps of emotion regulation, that is, difficulties in identifying and describing one's feelings and distinguishing between feelings and emotionally arousing body sensations (i.e., alexithymia) have also been reported in persons with FMS and also partly explain difficulties in the personal and social adaptation to the disease [62–64]. According to these results of previous investigations and the present study findings, changing the negative evaluation and intolerability of emotions, as proposed by cognitive-behavioral therapy, or promoting the acceptance of difficult emotions (and pain), which is a core outcome in acceptance and commitment therapy, might be crucial goals to improve adjustment in persons with FMS [65].

It is important to note that differences in all the aforementioned psychological mechanisms may enhance the understanding of FM as a heterogeneous entity on a dimensional spectrum but in which we can find identifiable profiles differentiated based on functionality and emotional impact [16]. Identifying these profiles could help in the development and design of more personalized and effective therapeutic options, prioritizing those therapeutic elements that work on psychological variables directly related to a better functionality in fibromyalgia.

Another important finding in our study was revealed after the binary logistic regression. Specifically, only five psychological variables (together with age, which was used as a covariate) significantly predicted group membership. These variables were harm, emotion, request, guarding, and task persistence. Consistent with the evidence from the literature on different pain populations [66–68], all these coping and beliefs variables were negatively related to functioning and emotional adjustment, with the exception of task persistence, in the sense that they were more present in cluster 1 (associated with worse mental health) compared to the high-functioning group (cluster 2). Overall, this finding supports past research in chronic pain populations showing that psychological mechanisms correlate with each other and have important commonalities [40], which means that only subsets of psychological variables are likely to contribute unique variance to the prediction of outcomes. This result is important because it points to the variables that might be more relevant for clinical purposes in relation to patient functioning, which could help to maximize the cost-effectiveness of interdisciplinary treatments. According to our findings, our results support the importance of three beliefs variables (i.e., harm, solicitude, and emotion) and two coping factors (i.e., guarding and task persistence), as they predicted 31% of the variance of group belonging and the inclusion of the remaining beliefs/coping strategies only added an additional 3% of explained variance.

The SOPA beliefs subscales of harm (i.e., the belief that pain is a sign of self-harm), solicitude (i.e., to think that others should pay attention to one's pain), and emotion (i.e., the belief that emotions influence one's experience of pain) have already been linked to greater severity of pain interference and poorer mental health in past research [69–71], which is consistent with our findings. Again in line with our results, guarding (i.e., to restrict the movement or general use of a body part) has been previously associated with more disability, pain interference, and psychosocial distress, as well as greater FM impact and worse functioning [72–74]. Finally, the task persistence subscale (i.e., to continue on doing an activity despite the pain) has been related with better psychological and physical functioning and lower levels of pain interference and disability [67, 75, 76], which is also consistent with our results. Novel to the literature, the present investigation presents a parsimonious set of predictors that appear to account for a considerable amount of variance in physical and mental functioning in persons with FM, which may be useful to personalize interventions according to the functioning status of patients. The management of beliefs and coping in psychotherapy has been traditionally approached from traditional cognitive-behavioral therapy with good results [77]. In recent years and largely due to the uncontrollability of chronic pain [78], other approaches based on third generation therapies have gained ground in the field of pain management. In particular, acceptance and commitment therapy, in which the goal is no longer to restructure or change one's beliefs but to modify one's relationship with such beliefs, has become particularly popular in the past years [72]. While our findings might be interpreted as more aligned with traditional forms of cognitive-behavioral therapy, identifying the beliefs with which one is more attached is also important for acceptance and commitment therapy (e.g., to take distance from them with cognitive defusion techniques). Therefore, the present study findings might also be relevant for clinicians interested in more modern forms of cognitive-behavioral therapy. In relation to coping, acceptance and commitment therapy does not impose the use of any coping strategy. As opposed to this, the psychological flexibility model in which acceptance and commitment therapy is grounded suggests that any behavior or coping strategy can be adaptive or maladaptive depending on the context in which it is performed (i.e., whether a context of acceptance, mindfulness, defusion with thoughts, self-as-context, committed action, and orientation to values occurs) [79]. Therefore, both guarding and task persistence could be adaptive or maladaptive depending on the context in which they are performed (e.g., whether they are performed in a context of psychological flexibility and contact with values or not). This explains why task persistence, when in an excessive and rigid manner, is associated with detrimental outcomes in persons with FMS [27]. In sum, those interested in the psychological flexibility model might find our findings in relation to coping useful in the sense that guarding might be seen as a form of some experiential avoidance, while task persistence might be viewed as a form of committed action in direction to something (a task) that is important to the individual.

In addition to the results with beliefs and coping, our study also evidenced that age was negatively related to FM impact and psychological distress. While the prevalence of pain increases with age [80], research has shown that pain is often perceived as less severe at older ages [81]. Thus, even though ageing and the associated body changes may lead to an increased risk of suffering persistent pain, it is possible that older individuals face pain in a healthier manner. For example, it is possible that pain is experienced as a more inevitable experience as age increases [82], which might lead to less frustration and more acceptance, thus opening the avenue for more adaptive forms of coping. What our findings suggest is again in the line of personalized interventions, in the sense that young people with FM might need more psychological help compared to older individuals with the disease.

The results presented in our study must be interpreted considering some limitations. First, the results apply to a specific population, that is, women diagnosed with fibromyalgia, so they cannot be generalized to males and other populations with chronic pain. In addition, some methodological limitations should be highlighted, such as the use of a correlation cross-sectional design, which prevents us from drawing temporal and causal interpretations of the findings. Finally, as we have relied exclusively on self-report measures, the data on functionality should be interpreted as subjective data, so the extent to which the present results are also true for objective functioning status is unclear. While this is of course a limitation and the inclusion of objective measures of functioning with wearable devices or sensors would be informative, it is also true that subjective appraisals are a fundamental part of the individual's experience, and even when it comes to pain perceptions, the measure of subjective pain has become much more popular and recommended than attempts to evaluate pain objectively [83].

Despite these limitations, the results of our work may have some clinical implications. Specifically, the present study has contributed to the identification of subgroups of patients with fibromyalgia according not only to general functioning but also to mental distress. Additionally, our results support the idea that a relatively small set of three cognitive (beliefs) and two coping variables is sufficient to predict group belonging (high vs. low functioning) in persons with FM. Overall, we have argued that this might be important to make interdisciplinary interventions more efficient by targeting the psychological mechanisms that differ the most when comparing functioning subgroups in persons with FM.

## Figures and Tables

**Table 1:** Cluster analysis of the participants.

	Cluster 1		Cluster 2		*t*	*p*	95% CI	*d*
	Mean	SD	Mean	SD				
Coping								
Guarding	3.71	1.62	2.75	1.61	**4.55**	<0.001	0.54, 1.37	0.59
Resting	4.64	1.68	4.10	1.73	**2.41**	0.017	0.10, 0.98	0.31
Ask for assistance	3.09	1.59	2.54	1.57	**2.65**	0.009	0.14, 0.95	0.34
Relaxation	3.44	1.94	3.56	1.97	−0.45	0.657	−0.62, 0.39	0.06
Task persistence	3.51	1.99	4.62	1.78	−**4.53**	<0.001	−1.60, −0.62	0.58
Exercise	2.43	1.90	3.14	2.47	−**2.45**	0.015	−1.27, −0.13	0.32
Seeking social support	2.48	1.65	2.52	2.68	−0.15	0.881	−0.61, 0.53	0.01
Self-statements	3.55	2.00	3.74	1.81	−0.74	0.460	−0.68, 0.31	0.09

Beliefs								
Control	1.61	0.98	1.90	0.95	−**2.28**	0.023	−0.53, −0.04	0.30
Disability	3.15	0.85	2.66	0.99	**4.05**	<0.001	0.25, 0.72	0.53
Harm	1.86	1.10	1.28	1.02	**4.25**	<0.001	0.31, 0.85	0.54
Emotion	3.01	0.90	2.55	1.10	**3.47**	0.001	0.20, 0.71	0.45
Solicitude	2.38	0.99	1.77	1.10	**4.45**	<0.001	0.34, 0.88	0.58
Medical procedures	1.96	0.99	2.15	0.93	−1.53	0.127	−0.44, 0.05	0.19
Fibromyalgia impact	74.41	10.40	50.72	15.50	**13.77**	<0.001	20.29, 27.07	1.79
Depression	31.16	7.99	14.73	6.03	**17.96**	<0.001	14.62, 18.23	2.32
Age	45.11	9.48	48.20	9.97	−**2.44**	0.015	−5.59, −0.60	0.31

In bold font, significant differences between clusters.

**Table 2:** The first step and final model in the backward binary logistic regression predicting cluster membership.

	B	*p*	95% CI
First model—all variables			
Coping			
Guarding	−0.25	0.054	0.61, 1.01
Resting	0.07	0.516	0.87, 1.32
Ask for assistance	−0.08	0.518	0.72, 1.18
Relaxation	−0.10	0.354	0.72, 1.12
Task persistence	0.15	0.095	0.97, 1.39
Exercise	0.05	0.615	0.87, 1.28
Seeking social support	0.03	0.798	0.83, 1.28
Self-statements	0.06	0.613	0.85, 1.31
Beliefs			
Control	0.21	0.297	0.83, 1.84
Disability	−0.26	0.173	053, 1.12
Harm	−0.37	0.034	0.49, 0.97
Emotion	−0.42	0.027	0.46, 0.95
Solicitude	−0.35	0.044	0.50, 0.99
Medical procedures	0.14	0.389	0.83, 1.60
Age	0.03	0.120	0.99, 1.06

Final model—selected variables			
Coping			
Guarding	−0.30	0.004	0.61, 0.91
Task persistence	0.18	0.031	1.02, 1.41
Beliefs			
Harm	−0.41	0.009	0.49, 0.90
Emotion	−0.35	0.047	0.50, 1.00
Solicitude	−0.32	0.044	0.53, 0.99
Age	0.04	0.029	1.00, 1.07

## Data Availability

The data used to support the findings of this study are included within the article and are available from the corresponding author upon request.
